# Comparison of staging MRI to re‐resection for localised bladder cancer: Narrative review

**DOI:** 10.1002/bco2.365

**Published:** 2024-04-24

**Authors:** Hugo Klempfner, Paul Anderson

**Affiliations:** ^1^ Department of Medicine, Melbourne Health and Northern Health The University of Melbourne Victoria Melbourne Australia; ^2^ Department of Urology Royal Melbourne Hospital Melbourne Australia

**Keywords:** bladder cancer, multiparametric MRI, narrative review, re‐TURBT, VI‐RADS

## Abstract

**Introduction:**

Bladder cancer (BCa) is characterised by high prevalence, multifocality, and frequent recurrence, imposing significant clinical and economic burdens. Accurate staging, particularly distinguishing non‐muscle‐invasive bladder cancer (NMIBC) from muscle‐invasive bladder cancer (MIBC) disease, is crucial for guiding treatment decisions. This narrative review explores the potential implications of incorporating multiparametric magnetic resonance imaging (mpMRI) and the Vesical Imaging Reporting Data System (VI‐RADS) into BCa staging, focusing on repeat transurethral resection of bladder tumour (re‐TURBT).

**Methods:**

A comprehensive search of PubMed, EMBASE, and MEDLINE databases identified studies published from 2018 to 2023 discussing mpMRI or VI‐RADS in the context of re‐TURBT for BCa staging. Studies meeting inclusion criteria underwent qualitative analysis.

**Results:**

Six recent studies met inclusion criteria. VI‐RADS scoring, accurately predicted muscle invasion, aiding in NMIBC/MIBC differentiation. VI‐RADS scores of ≥3 indicated MIBC with high sensitivity and specificity. VI‐RADS potentially identified patients benefiting from re‐TURBT and those for whom it could be safely omitted.

**Discussion:**

mpMRI and VI‐RADS offer promising prospects for BCa staging, potentially correlating more closely with re‐TURBT and radical cystectomy histopathology than initial TURBT. However, validation and careful evaluation of clinical integration are needed. Future research should refine patient selection and optimise mpMRI's role in BCa management.

**Conclusion:**

VI‐RADS scoring could revolutionise BCa staging, especially regarding re‐TURBT. There is potential that VI‐RADS correlates more with the histopathology of re‐TURBT and radical cystectomy than initial TURBT. While promising, ongoing research is essential to validate utility, refine selection criteria, and address economic considerations. Integration of VI‐RADS into BCa staging holds potential benefits for patients and health care systems.

**Abbreviations**
AUA: American Urological Association
AUC: area under the curve
BCa: bladder cancer
BCG: Bacillus Calmette‐Guerin
CIS: carcinoma in situ
CSS: cancer‐specific survival
CT: computerised tomography
CUA: Canadian Urological Association
EORTC: European Organization for Research and Treatment of Cancer
HR‐NMIBC: high‐risk non‐muscle‐invasive bladder cancer
MIBC: muscle‐invasive bladder cancer
mpMRI: multiparametric magnetic resonance imaging
MRI: magnetic resonance imaging
NAC: neoadjuvant chemotherapy
NCCN: National Comprehensive Cancer Network
NICE: National Institute for Health and Care Excellence
NMIBC: non‐muscle‐invasive bladder cancer
PRISMA: Preferred Reporting Items for Systematic and Meta‐analyses
RC: radical cystectomy
re‐TURBT: repeat transurethral resection of bladder tumour
TNM: Tumour, Node, Metastasis (staging system)
TUR: transurethral resection
TURBT: transurethral resection of bladder tumour
US: ultrasound
VI‐RADS: Vesical Imaging Reporting Data System

## 1 INTRODUCTION

### 1.1 bladder Cancer

Bladder cancer (BCa) is the ninth most diagnosed cancer worldwide, accounting for 3% of all cancer diagnoses with over 550 000 incidences in 2018,^1^ a notable increase from 430 000 in 2012.^2^ Its high recurrence rates and significant morbidity contribute to BCa having one of the highest lifetime treatment costs for all cancers.^3^

The bladder is a hollow pelvic organ that collects urine from the kidneys through the ureters and expels it via the urethra. The bladder has four layers:

1. The urothelium, a specialised pseudostratified columnar epithelium (transitional cells) that forms the internal lining mucosa and can flatten under pressure to accommodate the changing volume of urine.
2. The lamina propria, a highly vascularised connective tissue layer.
3. The muscularis propria, a smooth muscle layer (detrusor muscle).
4. The adventitia, the outermost connective tissue layer surrounded by perivesicular fat.^4^

Around 90% of BCa cases are urothelial carcinoma,^5^ an epithelial tumour from the transitional cells of the bladder mucosa. It is known for its multifocality and recurrence potential, with invasion of the muscularis propria a critical prognostic factor.^5^

### 1.2 bladder cancer diagnosis and staging

**FIGURE 1:**
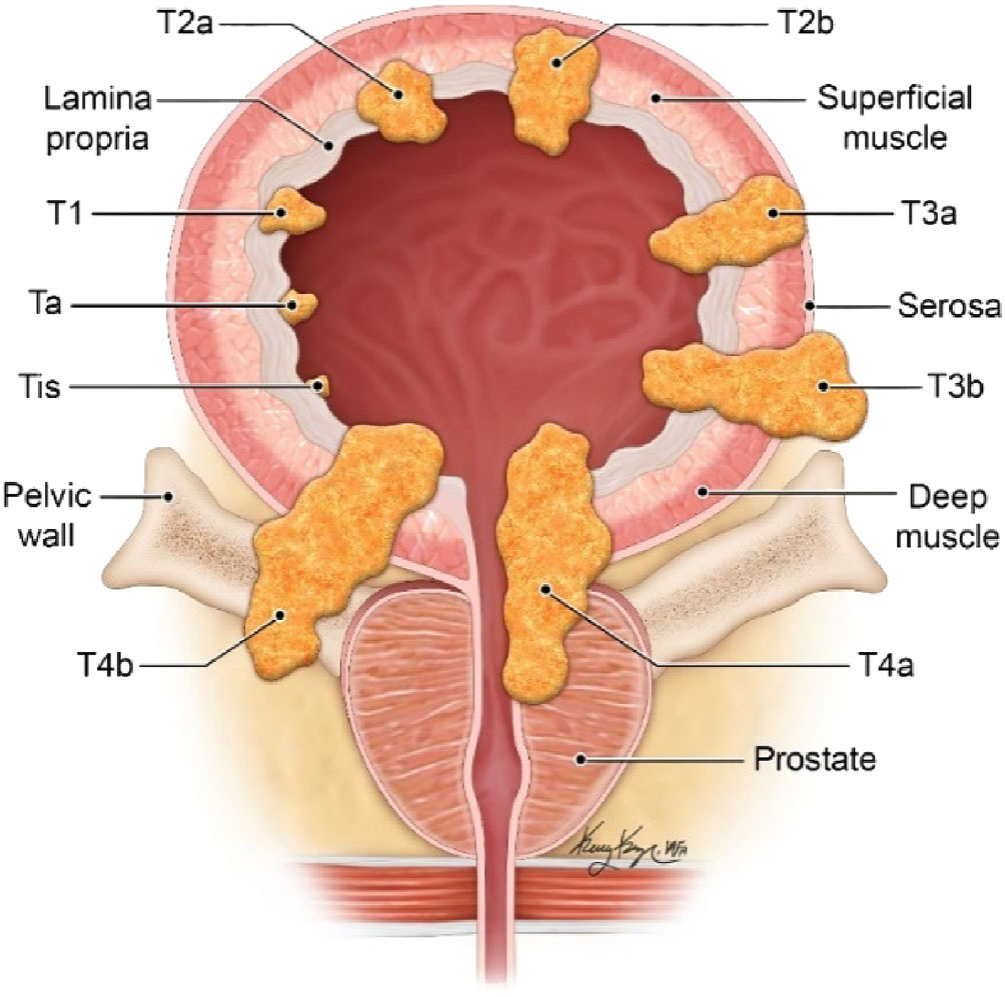
Diagram illustrating the layers of the bladder with the different T‐stages of bladder cancer.^7^

Initial BCa diagnosis relies on cystoscopy as the primary test, followed by a transurethral resection of bladder tumour (TURBT), to stage the disease based on histopathological investigation of the specimen.^6^ At this point in time, conventional imaging is unable to provide information for staging localised BCa. Tumour staging follows the TNM classification system. The T stage corresponds to the depth of invasion of the lesion (as seen in Figure 1):

- Tis is also known as carcinoma in situ (CIS) and is a “flat tumour.”
- Ta grows into the urothelium.
- T1 invades the lamina propria.
- T2 infiltrates the detrusor muscle, with T2a the superficial lining and T2b the deeper lining of the muscle.
- T3 extends past the detrusor into the outer serosa/perivesicular fat.
- T4 grows into the adjacent structures.

#### 1.2.1 Non‐muscle‐invasive bladder cancer (NMIBC) and muscle‐invasive bladder cancer (MIBC)

Distinguishing between NMIBC and MIBC is crucial during BCa staging, as it significantly influences treatment strategies, prognosis, and recurrence rates. NMIBC, encompassing Tis, Ta, and T1 BCa, accounts for about 75% of cases and boasts a high cancer‐specific survival (CSS) rate of 85%–98%.^8^ In contrast, the remaining 25% with MIBC face a significantly lower 5‐year CSS of approximately 50%–82%.^9^ Hence, assessing muscle invasion is paramount, shaping treatment plans.

NMIBC treatment typically involves TURBT with or without intravesical Bacillus Calmette‐Guerin (BCG) therapy, as well as possible radical cystectomy for patients with high risk of progression.^6^ For MIBC patients, radical cystectomy is the mainstay surgical treatment sometimes with neoadjuvant chemotherapy (NAC), immunotherapy, or radiotherapy.^10^

### 1.3 Repeat transurethral resection

There is a risk of understaging at initial TURBT, prompting guidelines to recommend a second resection (re‐TURBT) within 4–6 weeks to prevent undertreatment.^11^ Understaging can occur due to tumour morphology (such as tentacular spread or multifocality) or relating to technical inadequacies of the resection (such as incomplete resection or absence of muscle in specimen). Indications for re‐TURBT include T1 tumours, high‐grade tumours, or initial TURBT specimens lacking detrusor muscle.^6^ Re‐TURBT aims to avoid NMIBC understaging, remove residual BCa missed in the initial procedure, and provide additional prognostic information. Understaging of NMIBCs occurs around 7%–30% of the time, or up to 45% when detrusor muscle is not in initial TURBT.^12^, ^13^ For T1 tumours, upstaging to MIBC at re‐TURBT occurs 2.8% of the time,^14^ while only 0.4% in Ta tumours^15^; 31% of re‐TURBTs identify residual disease.^16^ Furthermore, the presence of residual disease at re‐TURBT is itself a prognostic factor that may indicate escalation of treatment. Additionally, re‐TURBT improves staging accuracy,^17^ clears residual disease,^14^ and improves the response to BCG therapy.^18^ However, it also imposes additional burdens on patients and health care resources and may delay radical treatment.

### 1.4 Multiparametric magnetic resonance imaging (mpMRI)

While traditional bladder imaging has limitations in local BCa staging, as differentiation between bladder layers is difficult on computerised tomography (CT) and ultrasound (US),^7^ mpMRI offers promise in this regard. Currently, it is unknown whether bladder MRI is sufficiently reliable to safely substitute re‐TURBT.

**FIGURE 2:**
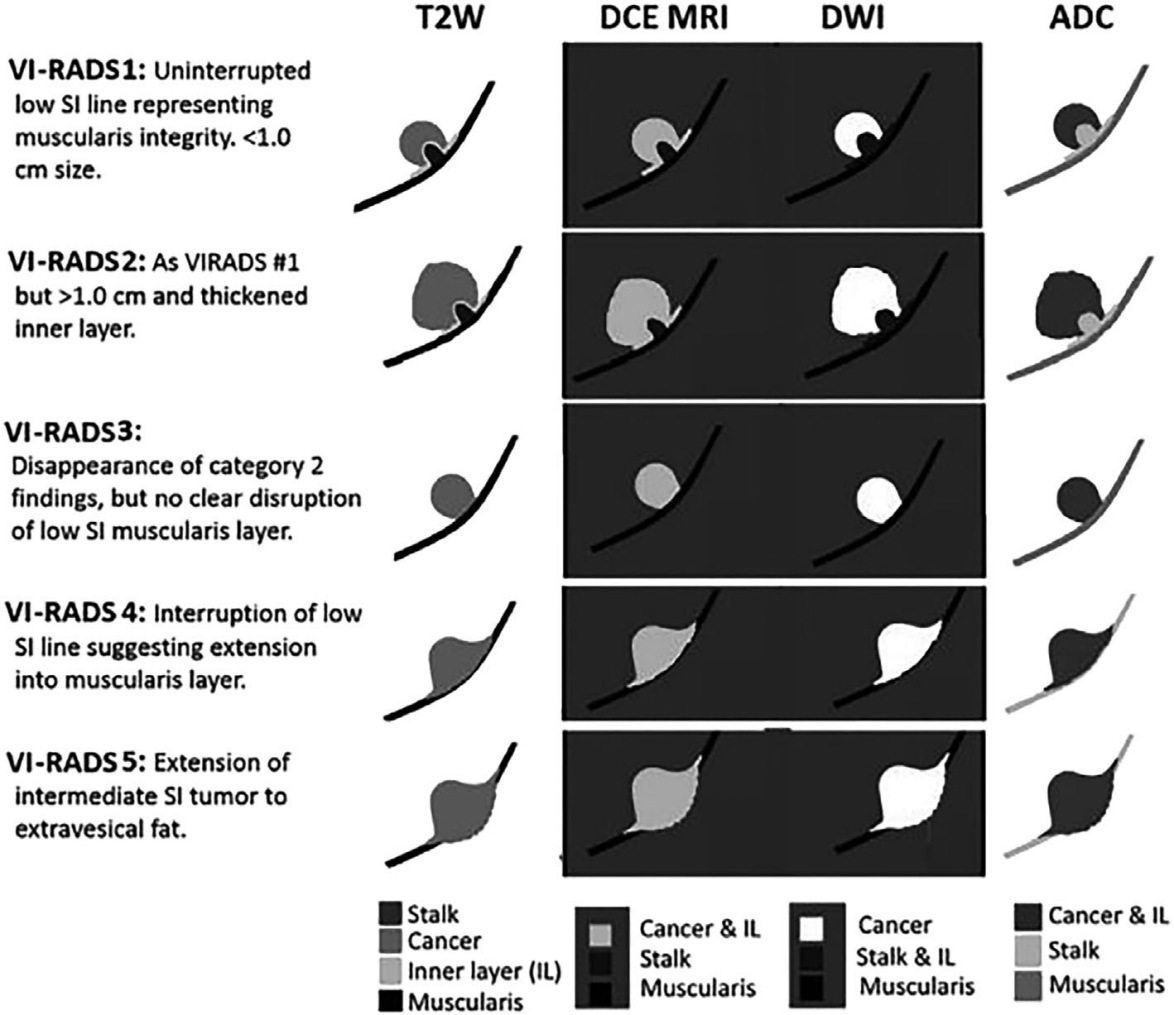
Scoring system for VI‐RADS.^20^

Inspired by the successful introduction of mpMRI via the Prostate Imaging‐Reporting and Data System for prostate cancer, the Vesical Imaging Reporting Data System (VI‐RADS) score was developed for BCa. VI‐RADS employs multiparametric MRI, incorporating T2‐weighted imaging (T2WI), diffusion‐weighted imaging (DWI), and contrast‐enhanced MRI (CE‐MRI), to predict detrusor muscle invasion.^19^ Uro‐radiologists using VI‐RADS assign a score from 1 to 5, indicating likelihood of muscle invasiveness. Scores of 1 and 2 signify a low chance of muscle invasion, 3 is considered equivocal, while 4 and 5 indicate a high likelihood. A strict protocol guides the mpMRI prior to TURBT^19^ (as seen in Figure 2). Subsequent studies, including retrospectives, prospective investigations, and meta‐analyses, have validated VI‐RADS as a reliable tool for identifying muscle invasion.^21^ To minimise false‐negatives, a VI‐RADS score of ≥3 is considered indicative of MIBC, with recent meta‐analyses reporting high sensitivity (0.83–0.92), specificity (0.82–0.90), and area under the curve (AUC) (0.93–0.94) ranges.^21^, ^22^, ^23^, ^24^, ^25^ mpMRI preceding TURBT could potentially lead to earlier MIBC recognition and treatment initiation.^26^ VI‐RADS may also serve as a non‐invasive post‐treatment monitoring and follow‐up alternative to cystoscopy for BCa.^27^

### 1.5 Aim of narrative review

With the development of VI‐RADS and emergence of research confirming its validity, the role of re‐TURBT in BCa management warrants exploration. mpMRI could potentially offer an opportunity to safely avoid re‐TURBT in low‐risk NMIBC cases, potentially reducing patient burden and health care resource utilisation. Furthermore, mpMRI could play a pivotal role in expediting radical cystectomy for MIBC, thereby addressing delays in treatment. This narrative review assesses the feasibility and implications of incorporating mpMRI, specifically VI‐RADS, into the local staging of BCa for patients requiring re‐TURBT, emphasising the potential paradigm shift in BCa management.

## 2 METHODS

### 2.1 Literature search

A comprehensive literature search was conducted using the online databases PubMed, Embase, and MEDLINE (Ovid) to identify relevant studies published between January 1st, 2018, and July 25th, 2023. The search strategy was developed based on the Patient‐Index test‐Comparator‐Outcome‐Study design (PICOS) criteria, with the primary research question formulated as follows: “How does magnetic resonance imaging (MRI) compare to repeat transurethral resection of bladder tumour (re‐TURBT) for bladder cancer staging?” Boolean operators were employed to combine the following search terms: “VI‐RADS” or “mpMRI,” “re‐TURBT,” and “Bladder Cancer.” Alternative related terms were also included to ensure comprehensive coverage of relevant literature, as seen in Appendix A.

### 2.2 Inclusion and exclusion criteria

Studies were included in this narrative review if they met the following criteria:

1. The study discussed the utilisation of mpMRI or VI‐RADS in the context of re‐TURBT.
2. The study was published in English.
3. The full text of the study was available for review.

Studies were excluded from consideration if they:

1. Were published prior to the introduction of VI‐RADS in 2018, as the development of this scoring system is considered a significant milestone in the field.
2. Did not specifically address the implications of VI‐RADS on repeat TURBT.

### 2.3 Study selection

The study followed the recommendations of Preferred Reporting Items for Systematic and Meta‐analyses (PRISMA) guidelines.^28^ Initially, the identified studies were screened based on titles and abstracts to determine their relevance to the research question. Subsequently, full‐text articles of potentially relevant studies were retrieved and assessed for eligibility against the inclusion and exclusion criteria outlined above.

Results were reported qualitatively, and no synthesis of data was completed.

## 3 RESULTS

**FIGURE 3:**
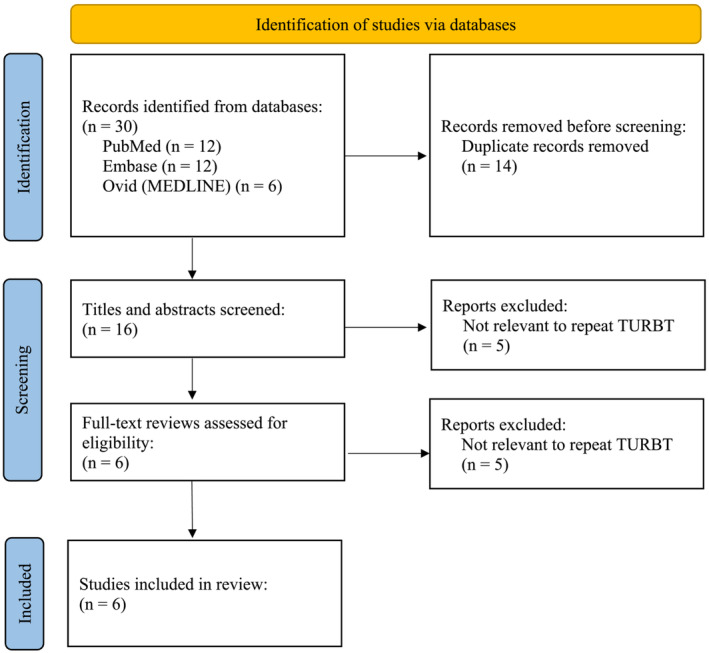
PRISMA diagram of literature search.

The comprehensive literature search conducted on PubMed, Embase, and MEDLINE (Ovid) using the specified search terms and criteria yielded a total of six articles for inclusion in this narrative review (Figure 3).

### 3.1 Result characteristics

Table 1 summarises the characteristics of the included studies, providing key information on study design, publication year, key findings, and conclusions of each study.

**TABLE 1:** Study results.

Study	Type of study	Participants	Results	Key conclusions
Del Giudice et al.^20^	Systematic review and meta‐analysis	2477 participants from 20 studies from 2019 to 2021	Sensitivity and Specificity were 0.87 and 0.86 for VI‐RADS cut‐off ≥3 while 0. and 0.94 for cut‐off ≥4	VI‐RADS cut‐off ≥3, given its broader inclusivity, delineates the shape of a predictive tool with better sensitivity, and would decrease the misdiagnosis of MIBC
Soria et al.^29^	Narrative review		*En bloc* resection improves the quality of resection. mpMRI may play a role in the selection of patients for re‐TURBT	Re‐TURBT is recommended internationally for HR‐NMIBC
Erkoc et al.^30^	Prospective study	330 patients with BCa suspicion underwent mpMRI and TURBT, of which 158 underwent reTURBT	Sensitivity, specificity, and AUC were 0.91, 0.92, and 0.900 for VI‐RADS cut‐off ≥3	Indirect association between lower VI‐RADS score and absence of residual disease after initial TURBT.
Jazayeri et al.^23^	Systematic review and meta‐analysis	2576 participants from 22 studies	AUC for VI‐RADS cut‐off ≥3 and ≥4 was 0.93 and 0.93. Sensitivity and specificity for VI‐RADS cut‐off ≥3 0.89 and 0.84.	Sources of inter‐study heterogeneity were the sample size >70, study design, single‐centre vs. multicentre, patient population characteristics (i.e., gender and age), reference standard, histology, magnetic strength, T2WI slice thickness, and the number of radiologists reporting the MRI results.
Taguchi et al.^31^	Algorithm proposal/narrative review		Patients with VI‐RADS ≤2 may not need to undergo sampling of the detrusor muscle nor second TURBT even if there is no muscle in the initial TURBT specimen, whereas patients with VI‐RADS ≥4 may skip conventional TURBT	Proposed algorithm enables the avoidance of unnecessary deep resection or second TURBT as well as delay to radical cystectomy
Bicchetti et al.^32^	Prospective study	139 patients with BCa suspicion underwent mpMRI and TURBT	Sensitivity of 93%–93%, specificity of 90%–92%, and AUC 0.95 for VI‐RADS cut‐off ≥3	In the multivariable logistic regression model, the VI‐RADS score, using both a cut‐off of 3 and 4, haematuria, tumour size, and concomitant hydronephrosis were the variables correlating with a bladder cancer staged as ≥T2.

Of the six selected articles, two were prospective studies, two were systematic reviews and meta‐analyses, and two were narrative reviews.

## 4 DISCUSSION

### 4.1 Bladder cancer staging

Staging is the single most important factor in determining bladder cancer treatment,^30^ serving as the cornerstone for treatment planning.^20^ Effective risk stratification tools, such as the European Organization for Research and Treatment of Cancer (EORTC) system, have been developed to guide therapeutic decisions.^33^ Nevertheless, it is imperative to recognise cases where existing tools may yield inconclusive results. NMIBCs constitute a heterogeneous group of tumours with varying risk profiles, which contribute to varying recurrence (30%–80%), progression (25%–50%), and cancer death within 5 years following bladder‐sparing treatment (16%–23%) rates.^34^ High‐grade MIBC poses a more significant threat, with a 5‐year overall survival of 68% following radical cystectomy, primarily due to an increased risk of metastatic disease.^35^

Distinguishing between NMIBC and MIBC is pivotal, as it profoundly impacts management strategies. TURBT serves both diagnostic and therapeutic roles in NMIBC but is solely a diagnostic procedure for MIBC patients.^36^ The quality of TURBT is contingent on the operator's skill and experience, influencing the presence of residual disease and requirement for re‐TURBT.^37^ VI‐RADS has been shown to have a high interreader reliability and agreement among radiologists, indicating less reliance on operator ability.^38^

Reliance on re‐TURBT for adequate staging results unintended consequences such as bladder perforation, delayed radical cystectomy,^39^ as well as financial implications, hospitalisation, and anaesthesia.^31^ Therefore, there is a need to look for alternative strategies.

### 4.2 High‐risk NMIBC

Classification of BCa into high risk or low risk is based on pathological staging and known factors.^40^ Invasion into the muscularis propria layer is paramount in the tumour node metastasis (TNM) staging system, classifying a BCa with mucosa invasion as T1 and muscularis propria invasion as T2. NMIBC, composed of Tis, Ta, and T1 BCa, is around 75% of BCa cases,^41^ and T2 stage or greater is MIBC. High‐risk NMIBC (HR‐NMIBC) encompasses T1 stage or Grade 3 tumours, recurrent cases, or Ta tumours larger than 3 cm.^30^

In the context of HR‐NMIBC, re‐TURBT is recommended to avoid understaging or residual tumour following initial TURBT. Currently, no diagnostic tools exist to stratify HR‐NMIBC patients' risk.^20^ Del Giudice et al.^20^ explored the potential of mpMRI in identifying HR‐NMIBC patients who may not necessitate re‐TURBT while also identifying those at risk of inadequate staging post‐TURBT. Their prospective study found that VI‐RADS score could be a potential predictor of adverse pathology at re‐TURBT, offering a possible means to select patients for whom re‐TURBT is necessary. However, caution is advised in advocating VI‐RADS as a tool for determining re‐TURBT necessity, as their study yielded a 31.5% rate of persistent NMIBC at re‐TURBT, consistent with the existing literature.^20^ Yet, their results are a promising foundation for future research focussing on the potential for mpMRI to identify high‐risk patients who may avoid re‐TURBT.

Strategies to improve TURBT technique have increased detrusor muscle inclusion in TURBT specimens. These improved techniques increase the quality of the specimens and the recurrence‐free survival,^16^, ^42^, ^43^, ^44^, ^45^ potentially reducing the rate of persistent NMIBC found. This improvement also enhances the safety of using VI‐RADS in informing re‐TURBT decision‐making.

### 4.3 Repeat TURBT

Prominent international urological and oncological guidelines, including those from the European Association of Urology (EAU), American Urological Association (AUA), National Institute for Health and Care Excellence (NICE), National Comprehensive Cancer Network (NCCN), and Canadian Urological Association (CUA), unanimously recommend a role for repeat TURBT in both diagnostic and therapeutic contexts.^29^ All recommend re‐TURBT for incomplete resection or the discovery of T1 tumour during initial TURBT, with some extending the recommendation to Ta tumours, or cases lacking muscle tissue in the initial specimen. Variations in recommendations exist, such as the EAU recommending re‐TURBT for tumours larger than 3 cm or those with multiple tumours.^6^

Soria et al.^29^ investigated the role of re‐TURBT in current NMIBC management and the feasibility of safely avoiding it in well‐selected patients. Recent trials employing the en bloc resection technique indicated a significant reduction in the risk of residual disease or upstaging after initial TURBT. The challenge lies in refining patient selection as re‐TURBT carries inherent morbidity, health‐care costs, and a risk of severe intraoperative and/or postoperative complications,^46^ often occurring while patients are still recovering from their initial TURBT. By improving selection, the overall number of re‐TURBTs can be reduced, but further research is required.

Determining the influence of residual NMIBC pathology on treatment decisions, Soria et al.^29^ concluded that re‐TURBT results should be integrated with established risk factors, mirroring current practice. Clinicopathologic prognostic factors, including tumour grade, stage, macroscopic appearance, carcinoma in situ (CIS), and the presence of detrusor tissue, have been associated with upstaging to MIBC and residual disease in TURBT specimens initially identified as NMIBC.^46^

A nomogram was developed to predict patients in whom re‐TURBT may be negative, allowing for its omission.^29^ For high‐grade T1 NMIBC patients with detrusor muscle in the initial TURBT, no CIS, and an en bloc TURBT, negative re‐TURBT is more likely. The en bloc technique independently predicts negative re‐TURBT outcomes, with a significantly lower rate of residual disease (6.4%)^47^ found in patients that had this technique compared with conventional methods (25%–50%).^13^ However, no tools currently exist for preoperatively or intraoperatively identifying the ideal re‐TURBT candidates among HR‐NMIBC patients.

While evidence shows that re‐TURBT improves detection of MIBC and residual disease, retrospective^48^ and prospective^49^ studies have shown limited impact on long‐term progression or CSS. Further, the presence or absence of detrusor in the initial TURBT specimen only minimally influences CSS for NMIBC patients.^50^

### 4.4 Utility of VI‐RADS

There are a limited number of studies relating VI‐RADS scores to re‐TURBT outcomes. One such study is that of Erkoc et al.,^30^ which reported that VI‐RADS sensitivity was highest when utilising histopathology from re‐TURBT or cystectomy specimens compared with initial TURBT results alone. This is possibly due to the high false‐negative rate of MIBC found in initial TURBT specimens. This suggests that VI‐RADS score may serve as a reliable indicator of re‐TURBT findings and correlate more closely with final BCa staging than initial TURBT results. Further research is needed to investigate whether discrepancies between initial resection and VI‐RADS staging should prompt re‐TURBT and whether histopathological staging concordant with VI‐RADS could justify omission of diagnostic re‐TURBT.

Del Giudice et al.^20^ also recognised the potential of VI‐RADS score derived from mpMRI prior to initial TURBT. Their findings suggest that VI‐RADS can be a strong tool for identifying patients with NMIBC who likely benefit from re‐TURBT. Furthermore, it can help identify patients for whom re‐TURBT may be unnecessary. This diagnostic value of VI‐RADS lies in its ability to characterise T1 and high‐grade Ta BCa, which might otherwise be under‐sampled or under‐staged.

Taguchi et al.^31^ proposed an innovative BCa management algorithm that leverages the high sensitivity of VI‐RADS ≥3 and the high specificity of VI‐RADS ≥4. In this algorithm, patients with a VI‐RADS scores of 2 or less could potentially avoid re‐TURBT, particularly when initial specimens lack detrusor muscle. On the other hand, patients with a VI‐RADS score of 4 or higher might proceed directly to radical cystectomy, circumventing the need for deep resection or re‐TURBT. This approach aims to reduce unnecessary procedures and associated risks for VI‐RADS ≤2 while expediting care for VI‐RADS ≥4. A similar algorithm to this is currently under evaluation in a randomised controlled study known as the BladderPath study.^51^ The preliminary results of the BladderPath study, although inconclusive at this stage, hint at a potential increase in false‐positive cases associated with the VI‐RADS‐based approach.^51^ Taguchi et al.^31^ suggest that due to the ease, reliability, and safety of MRI, it may be prudent for all BCa patients to undergo MRI before any other management.

Higochi et al.^52^ proposed a radical pathway primarily reliant on VI‐RADS scoring. mpMRI is the first‐line diagnostic measure and allocates patients to treatment based on their VI‐RADS score. MIBC receive radical cystectomy, NMIBC a TURBT. VI‐RADS 3 patients receive a diagnostic TURBT and then standard care.^52^ However the evidence for VI‐RADS does not yet support this level of conviction to its findings.

Bicchetti et al.^32^ explored the combination of VI‐RADS scores with other variables. Their findings indicated a VI‐RADS cut‐off of 3 and 4, haematuria, tumour size, and concomitant hydronephrosis correlated with BCa stages of T2 or greater at histopathology, resulting in the development of their own predictive pathway.

One significant advantage of undergoing mpMRI before TURBT is its ability to guide surgeons, radiologists, and pathologists in developing a more appropriate surgical strategy, which reduces the rate of re‐TURBT.^53^ Patients with BCa and VI‐RADS score ≥3 can have their lesions identified before cystoscopy. This allows surgeons to focus on the lesion during resection, potentially leading to a more successful removal. In HR‐NMIBC cases where the initial TURBT specimen lacks detrusor muscle or does not clearly indicate muscle invasion, re‐TURBT can be more targeted.^54^ A VI‐RADS score of 3 can also indicate to the surgeons that the degree of invasion is difficult to assess, prompting them to consider alternative approaches.^55^

While VI‐RADS holds promise, there are potential limitations. In specifics cases where VI‐RADS fails to detect muscle invasion, there is a risk of delayed diagnosis and management of MIBC.^30^ These patients may require two TURBTs, an mpMRI, and ultimately a radical cystectomy. Additionally, VI‐RADS relies on dynamic contrast‐enhanced MRI, which is not feasible in patients with contraindications to contrast agents. However, emerging research into bi‐parametric MRI assessments of BCa, which do not require contrast and have shorter image acquisition times, indicates comparable efficacy to mpMRI VI‐RADS scoring.^56^, ^57^ Further study is needed to validate this approach.

### 4.5 Future role of mpMRI

Soria et al.^29^ identified two potential cases where re‐TURBT could potentially be safely avoided. They are the two extremes of BCa: (1) MIBC necessitating immediate radical cystectomy and (2) low‐grade NMIBC requiring conservative treatment with intravesical immunotherapy. Future studies should focus on identifying patient and tumour characteristics to determine who can safely skip re‐TURBT.

Jazayeri et al.^25^ and Woo et al.^23^ highlighted that MRI characteristics such as magnetic strength and T2WI slice thickness potentially led to heterogeneity in their results. Scanner strength of 3 T had higher sensitivity than 1.5 T, as did a slice thickness of 2 or 3 mm compared with 4 or 5 mm.

Despite the development of numerous prognostic tools to predict NMIBC recurrence, response to therapy, or overall survival,^40^ none have incorporated clinical‐pathologic data with MRI markers such as VI‐RADS, tumour size, or hydronephrosis presence. Future research may yield predictive models that enable preoperative MIBC prediction, leading to a more personalised diagnostic pathway of either TURBT (intentionally without detrusor muscle) or radical cystectomy.

### 4.6 Limitations of narrative review

The limitations of this review include that it relies on a limited number of recent studies, potentially introducing research bias. Further interobserver variation of interpretation of scans and pathological specimens between centres may impact the generalisability of these techniques. Additionally, it does not explore the economic implications of mpMRI integration into clinical practice, which is crucial for health care policy considerations.

## 5 CONCLUSION

By analysing a selection of recent studies, it becomes evident that mpMRI, particularly through VI‐RADS, has the potential to significantly impact BCa management. The findings suggest that VI‐RADS scoring may serve as a valuable tool in identifying patients who may benefit from re‐TURBT and those for whom re‐TURBT could be safely omitted. Importantly, multiple studies have shown VI‐RADS has the potential to predict re‐resection or radical cystectomy pathology results. This could lead to more efficient treatment strategies, reduced patient burden, and optimised health care resource allocation. However, while the potential benefits are promising, further research and validation are required to refine patient selection criteria and define the role of bladder mpMRI in clinical practice.

## AUTHOR CONTRIBUTIONS

Hugo Klempfner and Paul Anderson contributed equally to the conception and design of the work, as well as the drafting and critical revision of the manuscript.

## CONFLICT OF INTEREST STATEMENT

The authors declare no conflicts of interest.

## Appendix A SEARCH STRATEGY

Embase + MEDLINE (Ovid):

Embase Classic + Embase <1947 to 2023 September 19>

Ovid MEDLINE(R) ALL <1946 to September 19, 2023>

**Table:**

#	Search	Results
1	(Repeated transurethral resection of bladder tumour or reTURB* or re‐TURB* or repeat* TURB* or second TURB* or restage* TURB*).mp. [mp = ti, ab, hw, tn, ot, dm, mf, dv, kf, fx, dq, bt, nm, ox, px, rx, ui, sy, ux, mx]	548
2	(mri, multiparametric or mris, multiparametric or multiparametric mri or multiparametric mris or multiparametric magnetic resonance imaging or mp‐mri).mp. or (exp Multiparametric Magnetic Resonance Imaging/ or Multiparametric Magnetic Resonance Imaging.mp.) or (vi‐rads.mp. or exp “vesical imaging reporting and data system”/or virads.mp.)	15 600
3	1 and 2	18
4	remove duplicates from 3	12

PubMed: Search: (((((((“repeated transurethral resection of bladder tumor”[All Fields]) OR (“repeated transurethral resections”[All Fields])) OR (reTURB*)) OR (re‐TURB*)) OR (repeat* TURB*)) OR (second* TURB*)) OR (restage* TURB*)) AND ((((((“mpmri”[All Fields]) OR (“virads”[All Fields])) OR (“vi rads”[All Fields])) OR (Vesical Imaging‐Reporting Data System)) OR (“multiparametric mri”[All Fields])) OR (“multiparametric magnetic resonance imaging”[All Fields])). 12 results
